# Functional Insight into the C-Terminal Extension of Halolysin SptA from Haloarchaeon *Natrinema* sp. J7

**DOI:** 10.1371/journal.pone.0023562

**Published:** 2011-08-19

**Authors:** Zhisheng Xu, Xin Du, Tingting Li, Fei Gan, Bing Tang, Xiao-Feng Tang

**Affiliations:** State Key Laboratory of Virology, College of Life Sciences, Wuhan University, Wuhan, China; University of South Florida College of Medicine, United States of America

## Abstract

Halolysin SptA from haloarchaeon *Natrinema* sp. J7 consists of a subtilisin-like catalytic domain and a C-terminal extension (CTE) containing two cysteine residues. In this report, we have investigated the function of the CTE using recombinant enzymes expressed in *Haloferax volcanii* WFD11. Deletion of the CTE greatly reduced but did not abolish protease activity, which suggests that the CTE is not essential for enzyme folding. Mutational analysis suggests that residues Cys303 and Cys338 within the CTE form a disulfide bond that make this domain resistant to autocleavage and proteolysis under hypotonic conditions. Characterization of full-length and CTE-truncation enzymes indicates the CTE not only confers extra stability to the enzyme but also assists enzyme activity on protein substrates by facilitating binding at high salinities. Interestingly, homology modeling of the CTE yields a β-jelly roll-like structure similar to those seen in Claudin-binding domain of *Clostridium perfringens* enterotoxin (clostridial C-CPE) and collagen binding domain (CBD), and the CTE also possesses collagen-binding activity, making it a potential candidate as an anchoring unit in drug delivery systems.

## Introduction

Haloarchaea thrive in hypersaline environments and generally require 15–30% NaCl as an optimal condition for their growth. Many of them secrete proteases to degrade protein substrates into small peptides and amino acids which the organisms then metabolize [1]. Halophilic enzymes, including proteases from haloarchaea, are active and stable at high salt concentrations, representing an attractive model for investigating enzymatic adaptation mechanisms at the molecular level [2]. Additionally, halophilic proteases show great potential as biocatalysts in the synthesis of oligopeptides in low water conditions (66%), including organic mixtures [3].

Many extracellular proteases have been isolated from haloarchaea, and almost all of them are serine proteases [1]. So far, the genes encoding the extracellular proteases 172P1 from *Natrialba asiatica* [4], R4 from *Haloferax mediterranei* [5], SptA from *Natrinema* sp. J7 [6] and Nep from *Natrialba magadii* [7] have been identified. Analysis of deduced amino acid sequences of these four enzymes indicate that they are members of the thermitase family of subtilisin-like serine proteases (subtilases) [8], and have been denoted as “halolysins” [1], [4]. To fulfill their physiological functions, halolysins should not only be stable in hypersaline environments, but also be efficiently active in spite of substrates undergoing salt-induced conformational change and precipitation. Exploring the molecular basis of halolysin activity at high salinities will provide new insights into the adaptation mechanisms of haloarchaea.

One common feature shared by halolysins is that the mature enzyme is composed of a subtilisin-like catalytic domain, and a C-terminal extension (CTE) with approximately 120 amino acid residues. CTEs have also been found in other subtilases, such as alkaline serine protease KP-43 from *Bacillus* sp. [9], hyperthermostable subtilisin Tk-SP from *Thermococcus kodakaraensis* [10], tomato subtilase 3 [11], kexin-like protease from *Aeromonas sobria* [12], yeast Kex2 [13] and mammalian furin [14]. The CTEs of these enzymes are proposed to be involved in enzyme stability and substrate recognition or binding. In addition, the precursor of aqualysin from *Thermus aquaticus* YT-1 has a C-terminal pro-sequence, which is required for enzyme secretion but is later processed to form the mature enzyme [15]. In haloarchaea, the CTE of halolysin R4 has been proposed to be functionally important, possibly by providing structural stability. However, halolysin R4 has proven difficult to purify and biochemically characterize in detail [5] and the function of haloarchaeal CTE domains is yet to be elucidated.

Halolysin SptA (formerly SptA protease) is extracellularly produced by *Natrinema* sp. J7 [6]. The SptA precursor is composed of a 49 residue signal peptide, a 103 residue propeptide, and a 413 residue mature region containing a CTE. When expressed in *Haloferax volcanii* WFD11, the recombinant SptA was secreted into the medium as an active mature form, and was stable enough to purify for further investigation [6]. In addition, mature SptA has four cysteine residues, two of which are conserved among halolysins and contained in the CTE. In this report, we perform deletion and point mutation analyses of the halolysin SptA C-terminal extension and discuss the possible roles of the CTE and its cysteine residues in enzyme stability, activity and substrate binding.

## Results

### Expression of C-terminal truncation mutants in *Hfx. volcanii*

**Figure 1:**
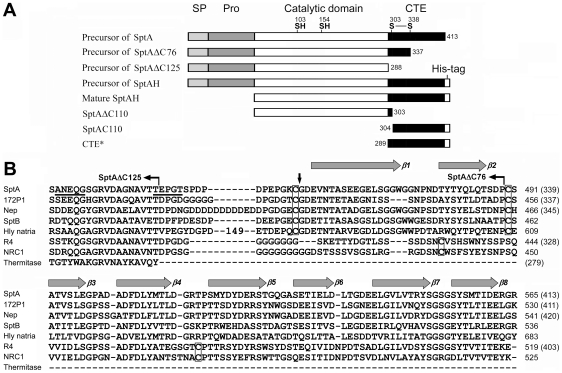
Schematic representation of the primary structures of SptA precursor and its derivatives (*A*) and amino acid sequence alignment of the CTEs of halolysins SptA (AAX19896) and SptB (AAX19897) from *Natrinema* sp. J7, 172P1 (P29143) from *Nab. asiatica*, Nep (AAV66536), a putative subtilisin (Hly natria) (ADD04299) from *Nab. magadii*, R4 (BAA10958) from *Hfx. mediterranei*, NRC1 (NP_281139) from *Halobacterium* sp. NRC-1, and thermitase (1THM_A) from *Thermoactinomyces vulgaris* (*B*). *A*, the signal peptide (SP), the N-terminal propeptide (Pro), the catalytic domain and the C-terminal extension (CTE) of SptA precursor are indicated. The locations of four cysteine residues are shown. The numbers on the right or left side of the boxes represent the positions of the C- or N-terminal residues of the proteins starting from the N-terminus of mature enzyme. *B*, the number (149) in the sequence (Hly natria) represents the number of inserted residues at the position indicated. The numbers on the right side represent the positions of the residues starting from the N-terminus of precursor or mature enzyme (in parentheses). Conserved cysteine residues are boxed. The vertical arrow indicates the cleavage site of the CTE of SptA under reducing conditions. The underlined sequence represents the N-terminal residues of the major autolysis products of SptA under lower salt conditions (Fig. 4A). The positions of C-termini of truncation mutants (SptAΔC125 and SptAΔC76) are indicated above the sequences. The eight β-strands (β1–8) in the CTE of SptA were predicted using DNAMAN software (Lynnon Biosoft Inc.).

**Figure 2:**
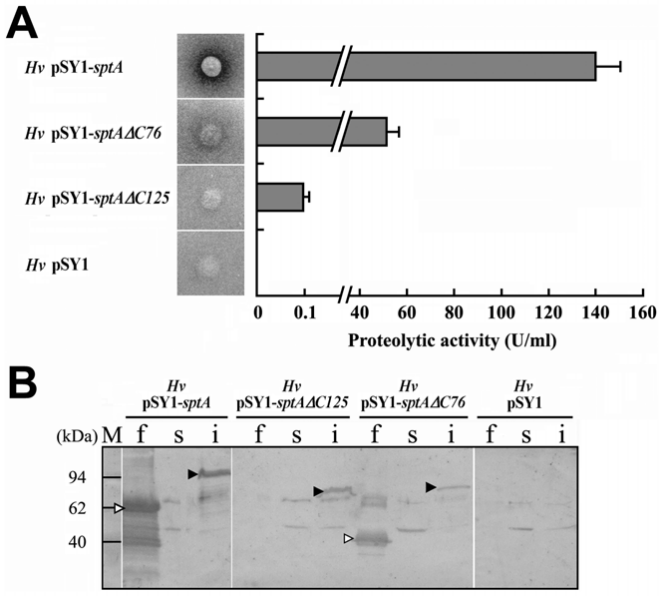
Expression of C-terminal truncation mutants of SptA in *Hfx. volcanii*. *A*, extracellular protease activity assay of *Hfx. volcanii* WFD11 (*Hv*) cells haboring different expression plasmids by detection of halo formation around colonies growing on skim milk plate (*left panel*), and activity assay of culture supernatants at late exponential phase using suc-AAPF-pNA as the substrate (*right panel*). *B*, immunoblot analysis of recombinant enzymes in culture supernatants (*f*), soluble (*s*) and insoluble (*i*) fractions of cells at late exponential phase. Pro- and mature forms of the enzyme were labeled with black and white arrow heads, respectively.

In order to probe the function of the CTE, we first constructed two truncation mutants, SptAΔC125 and SptAΔC76, by deleting 125 and 76 residues from the C-terminus of the enzyme, respectively (Fig. 1). As shown in Fig. 2A, hydrolysis halos could be detected around the colonies of *Hfx. volcanii* harboring the expression plasmid for SptA or SptAΔC76 growing on skim milk plates, but was hardly detected for SptAΔC125. This served as a means of detecting successful transformants as well as a basic indicator of activity. For a more robust activity assay, we used suc-AAPF-pNA as the substrate in reactions with culture supernatant containing secreted SptA, SptAΔC76, or SptAΔC125. Substantial levels of protease activity were found in culture supernatants individually expressing SptA and SptAΔC76, and trace activity was detected in that expressing SptAΔC125. In contrast, extracellular protease activity could not be detected in the control strain harboring plasmid pSY1, either by milk plate assay or enzyme activity measurement under the same conditions (Fig. 2A).

In order to identify the fractions containing the pro- and mature forms of the enzymes, we conducted immunoblot analyses. It should be mentioned that these recombinant enzymes migrate (Fig. 2B) higher than their theoretical molecular masses because they are acidic and resistant to SDS denaturation compositions, a common feature of halophilic enzymes [2]. Pro-forms of SptA, SptAΔC76 and SptAΔC125 were detected in the insoluble fractions but not in the soluble fractions of the cells (Fig. 2B). Mature forms of SptA and SptAΔC76 were found in culture supernatants, and mature SptAΔC125 could hardly be detected (Fig. 2B). This is consistent with our assay results that showed the activity of the culture supernatant containing SptAΔC125 was only approximately 1/1400 and approximately 1/500 of that containing SptA and SptAΔC76 (Fig. 2A), respectively. The appearance of other bands might result from the cross reaction between the antibodies and host proteins or/and degraded products of recombinant enzymes. Taken together, these results demonstrate that the CTE is important but not required for correct folding and activity of SptA.

### The protective effects of disulfide bond Cys303-Cys338

**Figure 3:**
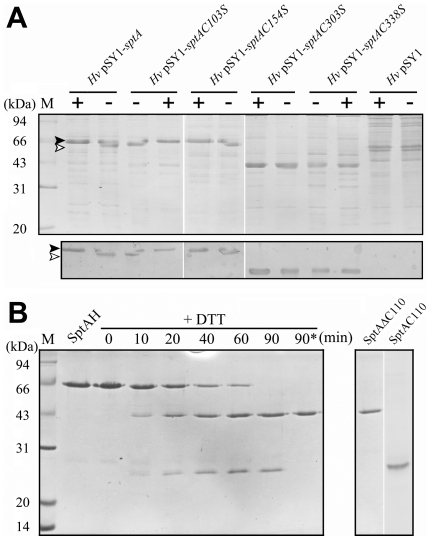
The effects of disulfide bond on the stability of SptA against the autocleavage of the CTE. *A*, detection of disulfide bond formation in SptA by SDS-PAGE analysis. The culture supernatants of *Hfx. volcanii* WFD11 (*Hv*) cells haboring different expression plasmids at late exponential phase were subjected to SDS-PAGE (*upper panel*) and immunoblot (*lower panel*) analyses in the presence (+) or absence (−) of 1% β-mercaptoethanol. Black and white arrow heads indicate the positions of reduced and non-reduced enzymes, respectively, on the gel. *B*, the processing of SptA under reducing conditions. Purified mature SptAH (0.1 µM) was incubated at 40°C in buffer A with 10 mM DTT. At the time intervals indicated, samples were taken and subjected to SDS-PAGE analysis (*left panel*). The lane denoted by an asterisk indicates the sample was first incubated at 40°C for 90 min, followed by heat treatment at 70°C for 5 min. The processed products, SptAΔC110 and SptAC110 (*right panel*), were purified as described in Materials and Methods.

Our next question regarding the function of the CTE focused on intramolecular disulfide bond formation, which is known to protect against autolysis in subtilisins [16] and is postulated for halolysin R4 [5]. The mature SptA contains four cysteine residues at positions 103, 154, 303 and 338. Of these, Cys303 and Cys338 are located within the CTE (Fig. 1). In order to identify the cysteine residues involved in disulfide bonds, mutants C103S, C154S, C303S and C338S were expressed in *Hfx. volcanii* WFD11 and the culture supernatants were subjected to SDS-PAGE under reducing and non-reducing conditions. C103S and C154S showed the same results as wild type SptA: in non-reducing conditions, they migrated faster than their reduced forms (Fig. 3A). This result indicates that the wild type, C103S, and C154S are all more compact in non-reducing conditions and have a smaller radius of gyration [17] than in reducing conditions. In contrast, no difference was observed in migration behaviors of C303S or C338S under reducing and non-reducing conditions. This finding suggests that in the wild type SptA, Cys303 and Cys338 formed the disulfide bond that was prevented in the C303S and C338S mutants.

Mature SptA, C103S and C154S in culture supernatants displayed the same apparent molecular mass of approximately 62 kDa, while C303S and C338S showed an apparent molecular mass of approximately 40 kDa (Fig. 3A). N-terminal sequencing identified the first 5 residues of the 40 kDa protein as YTPND, the same as those of mature SptA, suggesting that the C303S and C338S mutants underwent a C-terminal cleavage event. To further clarify this situation, a C-terminal His-tagged version of SptA (SptAH) was expressed in *Hfx. volcanii* WFD11, and was purified from the culture supernatant by Ni-NTA affinity chromatography (Fig. 3B). The purified SptAH displayed properties similar to SptA [6] in terms of salt dependence, activity and stability (data not shown), indicating that the presence of the His-tag had no significant effect on enzyme function. In the presence of reducing agents, SptAH gradually converted to the 40 kDa protein (named SptAΔC110), accompanied by the appearance of a 25 kDa protein (named SptAC110), which could be fully degraded after heat-treatment at 70°C for 5 min (Fig. 3B). The five N-terminal amino acid residues of SptAC110 were determined to be GDEVN, suggesting that the cleavage occurred between Cys303-Gly304 (Fig. 1). In addition, the C-terminal cleavage was not observed in PMSF-inactivated SptAH, but its CTE could be processed after treatment with active SptAH under reducing conditions (data not shown), implying the cleavage occurred in an intermolecular autocatalytic manner. These results demonstrate that disulfide bond Cys303-Cys338 plays an important role in stabilizing SptA against autocleavage of the CTE.

### The effect of the CTE on enzyme stability

**Figure 4:**
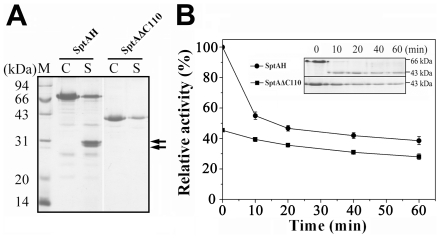
The effects of the CTE on the stability of SptA. *A*, autolysis of SptAH and SptAΔC110. The enzymes (5 µM) in 50 mM Tris-HCl (pH 8.0) containing 10 mM CaCl_2_ were incubated at 40°C for 3 h in the presence of 3 M (*lane C*) or 1 M NaCl (*lane S*), and then subjected to SDS-PAGE analysis. The two arrows indicate the major autolysis products of SptAH. *B*, heat inactivation profiles of SptAH and SptAΔC110. The enzymes (0.1 µM) in buffer A were incubated at 70°C for different time periods and then subjected to SDS-PAGE analysis (*inset*) and azocaseinolytic activity assay. Relative activities were calculated with the initial activity of SptAH before heat treatment defined as 100%. The values are expressed as means ± SD (*bars*) of three independent experiments.

To test the contributions of the CTE to enzyme stability, we first tested activity in low salt. At low enzyme concentrations (e.g., 0.1 µM), both SptAH and SptAΔC110 were very stable at 40°C in the presence of 3 M NaCl, and no loss of activity was observed after incubation at 40°C for 24 h. Meanwhile, the more concentrated enzymes (5 µM) showed slight autolysis at 3 M NaCl after incubation at 40°C for 3 h (Fig. 4A), suggesting only a small concentration dependence on autolysis. However, the two enzymes showed significant autolysis under lower salt conditions (e.g., 1 M NaCl), wherein SptAH could convert to two major autolysis products with apparent molecular mass of approximately 30 kDa (Fig. 4A). By N-terminal sequencing, the first five amino acid residues of the two products were identified as TEPGT and TSPDP, which coincided with the amino acid sequence of SptA after Thr287 and Gly291, respectively (Fig. 1). It was observed that the two products could bind to Ni-NTA affinity column (data not shown), implying the His-tag fused at the C-terminus was not processed. Therefore, they represent two forms of undigested CTE that are more resistant to proteolysis than the catalytic domain at low salinities. High temperatures were also used to test the stability limits of the enzymes and heat inactivation profiles of SptAH and SptAΔC110 were examined at 70°C in 3 M NaCl (Fig. 4B). Results showed that SptAH autocleaved its CTE and lost its azocaseinolytic activity faster and to a greater degree than SptAΔC110, which suggests that the CTE is important to activity.

**Figure 5:**
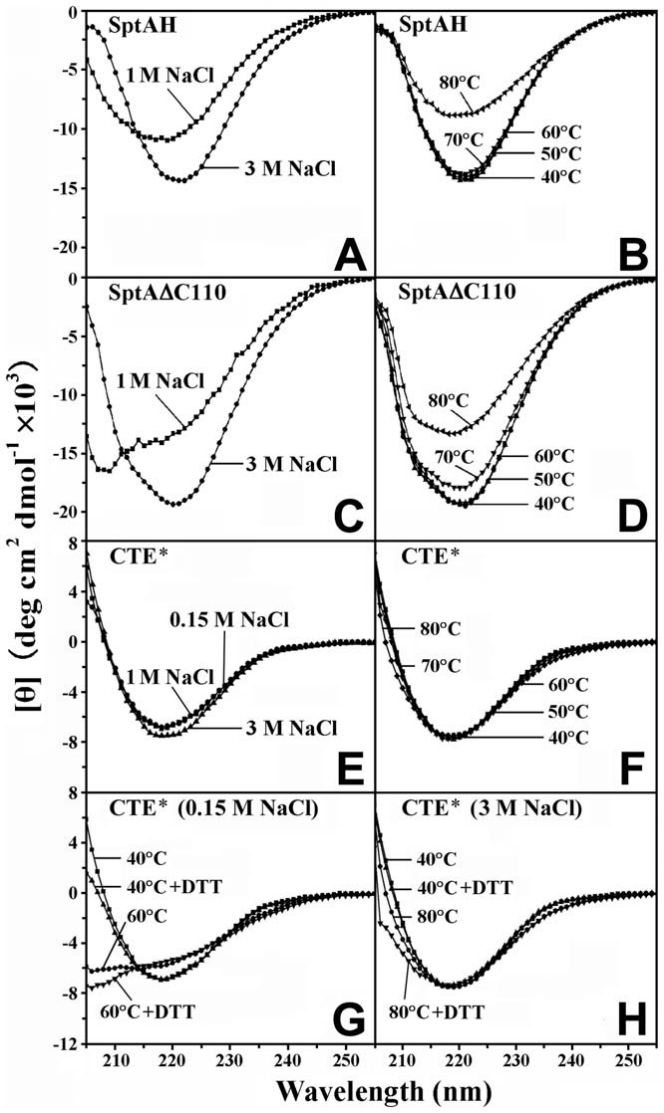
The far UV CD spectra of SptAH, SptAΔC110 and CTE*. The measurements were performed at a protein concentration of 0.2 mg/ml, either at 40°C in the presence of different concentrations of NaCl (*A*, *C*, and *E*), or at different temperatures in the presence of 3 M NaCl (*B*, *D*, and *F*), as described in Materials and Methods. In some cases (*G* and *H*), the spectra were recorded in the presence of 1 mM DTT.

The CTE interacts with the subtilisin domain via hydrophobic residues and these contacts have been proposed to provide stabilization to the enzymes [10], [14]. To test this hypothesis, we used CD to investigate CTE contributions to enzyme structure in solution. Far-UV CD spectra of SptAH, SptAΔC110 and CTE* were measured at different salinities or temperatures (Fig. 5). At 40°C, the mean residue ellipticity value of SptAH or CTE* is lower than that of SptAΔC110 in the presence of 3 M NaCl (Fig. 5A, C and E), as expected from the contributions of the β-strands in the CTE (Fig. 1) (β-strands show lower ellipticity than α-helices) [18]. When the salinity was decreased to 1 M or the temperature increased to 70°C, SptAΔC110 showed a larger global change in secondary structure than CTE* (Fig. 5C to F), indicating that the CTE domain is more stable than the catalytic domain. In contrast to SptAΔC110, both SptAH and CTE* showed no significant change in secondary structure in the temperature range of 40–70°C (Fig. 5B, D and F), demonstrating that the CTE domain, in addition to being stable itself, helps the stability of the catalytic domain. At elevated temperatures, CTE* exhibited a more significant change in CD spectrum under reducing conditions than under non-reducing conditions (Fig. 5G and H), suggesting that disulfide bond Cys303-Cys338 confers additional stability to the CTE domain.

### The importance of the CTE in enzyme activity

**Figure 6:**
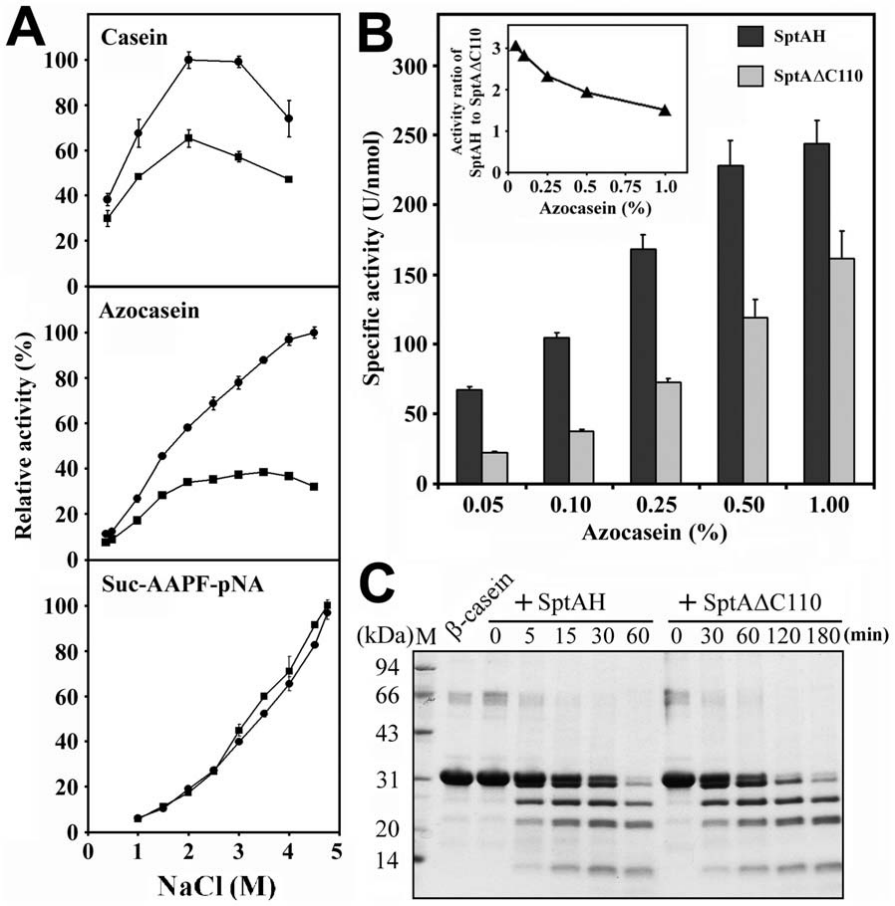
The effects of the CTE on enzyme activity of SptA. *A*, salt dependence of enzyme activity towards casein, azocasein or suc-AAPF-pNA. At different concentrations of NaCl, the activity of SptAH (•) and SptAΔC110(▪) was measured at 40°C as described in Materials and Methods. The values are expressed as means ± SD (*bars*) of three independent experiments. Maximal activity was taken as 100%. *B*, comparison of the activities of SptAH and SptAΔC110 at different concentrations of azocasein. The enzyme activity was measured at 40°C and 3 M NaCl, as described in Materials and Methods. The values are expressed as means ± SD (*bars*) of three independent experiments. The *inset* shows the SptAH/SptAΔC110 activities ratio at different substrate concentrations. *C*, digestion patterns of β-casein cleaved by SptAH and SptAΔC110. The reaction was carried out at 40°C in buffer A containing 60 µg/ml of β-casein (Sigma) and 5 nM of enzyme for different time periods, and then subjected to Tricine-SDS-PAGE analysis.

It was previously shown that SptA is most active on 0.5% azocasein in the presence of 2.5–3.0 M NaCl [6]. We investigated the salt dependence of our enzyme constructs to ascertain if the CTE assists the cleavage reaction. Using casein (1%) as the substrate, SptAH was most active at 2.0–3.0 M NaCl (Fig. 6A). During the course of our experiments, we noticed that the azocasein (0.5%) and casein (1%) solutions became turbid in the presence of more than 4 M and 3 M NaCl, respectively. In addition, it was found that optimal salt concentrations for enzymatic activity increased when substrate concentration decreased (data not shown), which may reflect a possible problem with substrate solubility at high salinities [19]. To reduce these effects, the salt dependence of SptAH and SptAΔC110 activities on azocasein were determined at a lower substrate concentration (0.25%). As shown in Fig. 6A, SptAH and SptAΔC110 displayed maximal azocaseinolytic activity above 4.5 M NaCl and at 3.5 M NaCl, respectively. Notably, SptAH showed higher azocaseinolytic activity than SptAΔC110 over the whole range of NaCl concentrations tested (0.5–4.5 M), and the difference became more pronounced as the salinity increased (Fig. 6A). In contrast, no significant difference was observed between the two enzymes in terms of their activities against the small synthetic substrate suc-AAPF-pNA, and these activities increased with rising salinity up to 4.75 M (Fig. 6A). A similar result was observed when suc-AAPL-pNA was used as an alternate small substrate (data not shown). The variation in enzymatic salt dependence toward the two sizes of substrates may be due differing levels of salt-induced substrate conformation changes, which would be more significant in proteins than in peptides [20]. In order to determine whether the SptA catalytic domain and CTE can act *in trans*, we tested equimolar mixtures of SptAΔC110 and SptAC110. These showed a lower level of azocaseinolytic activity similar to that of SptAΔC110 alone (data not shown), implying the covalent link between the two domains is necessary for the higher activity of wild-type SptA.

SptAH and SptAΔC110 showed similar kinetic parameters for the hydrolysis of suc-AAPF-pNA (Table 1), indicating the CTE has no significant effect on the enzyme's affinity for the small synthetic substrate. Kinetic characterization with protein substrates is difficult because these substrates contain more than one hydrolysable peptide bond. Nevertheless, the evidence that the SptAH/SptAΔC110 activities ratio increased as the substrate (azocasein) concentration decreased (Fig. 6B) implies SptAH has a higher affinity for the substrate than SptAΔC110. In addition, β-casein digestion products of both enzymes were the same (Fig. 6C), suggesting the CTE domain does not contribute significantly to the cleavage specificity of the enzyme towards casein. These results demonstrate that the CTE is beneficial for catalytic efficiency of SptA toward the larger protein substrate, most likely by facilitating the binding of the protein for catalysis.

**Table 1:** Kinetic parameters of SptAH and SptAΔC110 toward suc-AAPF-pNA.

Enzyme	*K*_m_ (mM)	*k*_cat_ (s^−1^)
SptAH	0.90±0.02	1462±210
SptAΔC110	0.92±0.01	1330±191

The values are expressed as the mean ± SD of three independent experiments.

### Binding of the CTE to insoluble substrates

**Figure 7:**
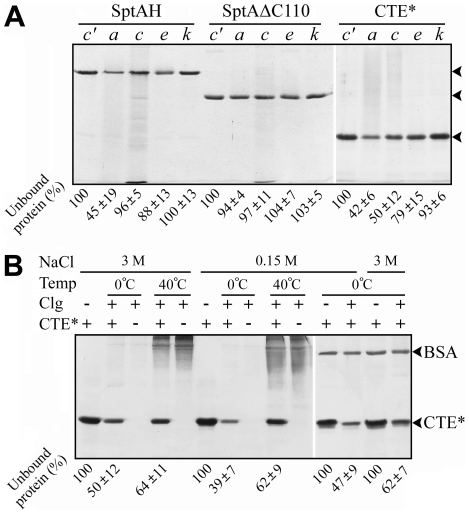
Binding capacities of the CTE toward insoluble substrates. *A*, binding of SptAH, SptAΔC110 and CTE* toward different insoluble substrates at 3 M NaCl. The proteins (15 µg/ml) were incubated at 0°C for 60 min in the absence (*c′*) or presence of 5 mg of azocoll (*a*), type I collagen (*c*), elastin-orcein (*e*) or keratin-azure (*k*). After centrifugation, the supernatants were subjected to SDS-PAGE analysis as described in Materials and Methods. The arrows indicate the positions of SptAH, SptAΔC110 and CTE* on the gel, respectively. *B*, binding of CTE* toward insoluble type I collagen (Clg) at different temperatures and salinities. The CTE* (15 µg/ml) was incubated at 0°C or 40°C for 180 min in buffer A (3 M NaCl) or buffer B (0.15 M NaCl), in the absence (−) or presence (+) of 5 mg of Clg. In some cases, BSA (10 µg/ml) was added into the binding mixture as a control. Numbers given below the gels indicate the densitometric ratios of unbound proteins compared with that of the control lane, and the values are expressed as means ± SD of three independent experiments.

In order to further ascertain the contribution of the CTE to enzymatic catalysis, and to investigate possible CTE binding interactions with substrates, insoluble azocoll, type I collagen, elastin-orcein and keratin-azure were employed for activity and binding assays. The activity assay results showed that, while both enzymes were inert towards elastin-orcein and keratin-azure, SptAH showed a higher activity (18.5±0.9 U/nmol) than SptAΔC110 (11.5±1.0 U/nmol) toward azocoll at 3 M NaCl, confirming the importance of the CTE in enzyme activity. The binding assays were carried out at 0°C to prevent substrate breakdown as much as possible [21]. In the presence of 3 M NaCl, SptAH significantly bound to azocoll, and showed a weak binding capacity towards elastin-orcein. In contrast, SptAΔC110 displayed no binding capacity for the four substrates tested (Fig. 7A), implying that the CTE alone is involved in the binding of the enzyme to these substrates. To test this hypothesis, CTE* was employed for binding assay. As shown in Fig.7A, the binding capacities of CTE* towards azocoll, elastin-orcein and keratin-azure were similar to those of SptAH. However, CTE* differed from SptAH in that about 50% of CTE* could bind to insoluble type I collagen at 3 M NaCl, while most of SptAH remained in the supernatant under the same conditions. Since various smaller polypeptides were detected in the supernatant of the sample of SptAH but not in that of CTE* (Fig.7A), the difference in type I collagen-binding behaviors between the two proteins was most likely due to partial hydrolysis of the insoluble collagen by SptAH and subsequent products release into the supernatant. CTE* was also able to bind to insoluble type I collagen at low salinities (e.g., 0.15 M NaCl), and its collagen-binding capacity was not significantly affected by the addition of BSA (Fig. 7B). At 40°C, the amount of unbound CTE* slightly increased, probably due to partial solubilization of the substrate as evidenced by the occurrence of background signals in the lanes (Fig. 7B).

### Conserved tertiary structure of the CTE

There has been no reported crystal structure of any halolysin so far. By using secondary structure prediction methods, eight β-strands could be found in the CTEs of SptA and other halolysins (Fig. 1). This was supported by the CD analysis of CTE*, which displays a spectrum typical of a β-strand backbone (Fig. 5). In addition, automated homology modeling of the SptA CTE yields a β-jelly roll-like structure (Fig. S1) that suggests a possible distant relationship to clostridial C-CPE (∼17% identity) and CBD (∼16% identity) domains. Although the similarity is low at the sequence level, the three domains have similar β-jelly roll-like structures and some key residues of clostridial C-CPE and CBD involved in receptor or collagen binding are conserved in the SptA CTE. For example, three Tyr residues near the extreme C-terminus of the C-CPE are critical for receptor binding [22], among them the residue Tyr310 is conserved in the SptA CTE (Tyr404) (Fig. S1B). In the case of clostridial CBD, three (Tyr970, Leu992 and Tyr996) of the five residues showing >5-fold reduction in collagen-binding activity upon mutation are present in the SptA CTE (Tyr368, Leu394 and Tyr398) (Fig. S1B). Moreover, three residues (Arg929, Phe952 and Val978) of clostridial CBD were shown to enhance collagen-binding activity upon mutation to Ala [23]. Interestingly, two Ala residues (Ala350 and Ala377) exist naturally in the SptA CTE at the positions corresponding to Phe952 and Val978 in clostridial CBD (Fig. S1B). Our results suggest that the CTE domain of SptA also has the ability to bind to collagen. We speculate that the binding of the CTE to substrates may be in a manner similar to the cases of clostridial CBD and C-CPE because of their structural similarity.

## Discussion

Downstream of the subtilisin-like catalytic domain, halolysins 172P, R4, Nep and SptA all have a respective CTE containing two cysteine residues (Fig. 1). The pioneer work of Kamekura et al. [5] suggested that the removal of the CTE from halolysin R4 abolished protease activity in culture supernatant of recombinant *Hfx. volcanii* WFD11 and, thus, the CTE has been proposed to provide an essential (but as yet unknown) function. In the case of SptA, although the deletion of its CTE led to a sharp decrease in protease activity in culture supernatant of *Hfx. volcanii* WFD11 expressing the mutant SptAΔC125, the appearance of active enzyme in the culture supernatants indicates the CTE is not essential for the folding of SptA. Since the amount of the secreted active SptAΔC125 is rather low relative to that of wild-type enzyme, we cannot exclude the possibility that the CTE may assist in the correct folding of the enzyme or/and its secretion. It is observed that inactive pro-form of SptAΔC125 was detected in the insoluble fraction of the recombinant cell. This is unlikely due to a lack of the CTE, because wild-type pro-SptA also existed in the insoluble fraction. Further investigation of the mechanisms of folding and secretion of the enzyme is required to find possible explanation for this observation.

Many subtilases contain disulfide bond(s) that contribute to enzyme stability [8]. For halolysins, it has been reported that the substitution of Cys316 and Cys352 within the CTE of halolysin R4 decreased enzyme stability in hypotonic solutions, possibly owing to disruption of potential disulfide bonds or perturbation of calcium binding site(s). However, the stabilization mechanism of the two cysteine residues has not yet been elucidated because of the difficulty of enzyme purification [5]. In this report, the experiments of Cys→Ser mutations clearly indicate that Cys303 and Cys338 form a disulfide bond in the CTE of SptA, and disruption of the disulfide bond results in the autocleavage of the CTE. The two cysteine residues within the CTE of SptA are conserved in 172P1, Nep and SptB, while those of R4 are conserved in halolysin NRC1 (Fig. 1). Despite variation in their locations, all these cysteine residues reside in the loop regions connecting the secondary structure elements (Fig. 1). The formation of a disulfide bond, as observed in SptA, can endow the CTE with structural stability against proteolysis, which seems to be a common stabilization strategy shared by halolysins.

The presence of the CTE domain increases the affinity of SptA for larger protein substrate rather than smaller peptide substrate. One reasonable explanation for this is that the CTE domain is situated too far away from the catalytic cleft to participate in the binding of small substrate (Fig. S1A). Conversely, the CTE domain may provide additional binding site(s) for protein substrate, and thus be beneficial to hydrolysis activity. However, the CTE domain does not contribute significantly to the cleavage specificity of SptA towards casein, unlike the cases of kexin-like proteases in which the P-domains are not only involved in substrate binding but also play an important role in the regulation of the substrate specificity of these enzymes [12], [24]. This distinction may be rationalized by consideration of the differences in physiological function and enzyme structure between SptA and kexin-like proteases. The members of kexin/furin family function as pro-protein processing proteases (convertases), and differ from other subtilases in that they have a high degree of specificity for cleavage after dibasic or multi-basic residues [8]. At the C-terminus of the P-domain in the kexin-like protease from *A. sobria*, there is an extra occluding region situated close to the active site, which may act as a steric obstacle and is important for substrate recognition (Fig. S1A) [12]. Unlike these convertases, halolysins serve as degradative proteases to provide haloarchaea with small peptides and amino acids as nutrition [1]. The lack of the occluding region is favorable for SptA to have broad substrate specificity, thereby allowing the enzyme to deal with different protein substrates in the environment. Given that SptA is produced by a haloarchaeon adapted to extremely saline environments, the role of its CTE domain in substrate binding seems to be of physiological importance. At high salt concentrations, proteolysis of protein substrates may be affected by limited accessibilities and availabilities of the substrates due to salt-induced conformation changes and precipitation (“salting-out” effect). Obviously, the enhanced substrate affinity afforded by the CTE domain would be advantageous for halolysins to fulfill their physiological roles at high salinities.

Recently, several crystal structures of subtilases with CTEs have been determined. Although their CTEs have very low or almost no sequential homology, they nonetheless fold into a β-jelly roll-like structure, such as the P-domains of mammalian furin [14], yeast Kex2 [13] and kexin-like protease from *A. sobria* [12], the C-domain of the protease KP-43 from *Bacillus* sp. [9], the Fn III domain of tomato subtilase 3 [11], as well as the β-jelly roll domain of subtilisin Tk-SP from *T. kodakaraensis* [10]. Additionally, fervidolysin from *Fervidobacterium pennivorans* [25] and streptococcal C5a peptidases [26], [27] possess two and three β-jelly roll domains at their C-termini, respectively. β-jelly roll-like structures are common in substrate or receptor-binding domains for other bacterial proteins, such as clostridial C-CPE [22] and CBD [23]. It is interesting that the CTE domain of SptA has the ability to bind to collagen at low salinities (e.g., 0.15 M NaCl). CBD has been well studied in mammalian matrix metalloproteinases and bacterial collagenases [28], but has not yet been reported in archaea. A large focus of protein engineering is the creation of molecules that bind to and enhance the delivery of therapeutics across the dermal barrier. The clostridial CBD has been used in a drug delivery system by acting as an anchoring unit to collagen, the major constituent of extracellular matrix of animals [29]. Recently, the CBD of the collagenase from thermophile *Geobacillus collagenovorans* MO-1 has been proposed to have the potential for applications to such a drug delivery system by taking advantage of the nonpathogenicity of the strain [21]. Likewise, the collagen-binding capacity of the CTE domain from a non-pathogenic haloarchaeon makes it a potential candidate for clinical applications. From a practical view point, durability and sustainability are highly desired for drug delivery systems. The apparent stabilization effect of the disulfide bond Cys303-Cys338 on the CTE domain of SptA might provide a rational basis for improving the stability of the anchoring unit.

## Materials and Methods

### Strains and growth conditions

*Natrinema* sp. J7 was isolated from a salt mine in China [6], and has been deposited in the China Center for Type Culture Collection (CCTCC) under the accession number AB91141. *Hfx. volcanii* WFD11 was used as the host for expression. These two halophilic archaea were cultured as described previously [6]. *Escherichia coli* JM110 and *E. coli* BL21(DE3) were used as the hosts for routing cloning and expression, respectively, and were grown at 37°C in Luria-Bertani (LB) medium supplemented with ampicillin (50 µg/ml) or kanamycin (30 µg/ml) as needed.

### DNA manipulation, plasmid construction and mutagenesis

The genomic DNA of *Natrinema* sp. J7 was prepared according to the method of Kamekura et al. [4], and was used as the template for PCR. The primers used in this study are listed in Table S1. The gene of wild type SptA was amplified with primers sptA-ATG1 and sptA-R1. The genes encoding the C-terminal truncation mutants (SptAΔC125 and SptAΔC76) and the C-terminal His-tagged version of halolysin SptA (SptAH), were amplified from the genomic DNA with the primer sptA-ATG1 in combination with the primers sptAΔC125-R, sptAΔC76-R, and sptAH-R, respectively. Overlapping PCR was employed as described previously [16] to introduce the C103S or C154S mutations into the SptA gene. Briefly, the 5′ end of the gene was amplified with the primer sptA-ATG1 in combination with the primer sptAC103S-R or sptAC154S-R, and the 3′ end of the gene was amplified with the primer sptA-R1 in combination with the primer sptAC103S-F or sptAC154S-F, respectively. The “megaprimer” method of site-directed mutagenesis [30] was used for substitutions C303S and C338S, with primers sptAC303S-F and sptAC338S-F used as the mutagenic primers. The amplified genes were ligated into the *Nde*I-*Nco*I restriction site of pSY1 [6] to construct the expression plasmids pSY1-*sptA*, pSY1-*sptAH*, pSY1-*sptAΔC125*, pSY1-*sptAΔC76*, pSY1-*sptAC103S*, pSY1-*sptAC154S*, pSY1-*sptAC303S* and pSY1-*sptAC338S*, respectively. This new expression plasmid pSY1-*sptA* for wild type SptA differs from the previously constructed pSPTA1 [6] in that the latter contains additional 104 nucleotides downstream from the stop codon. All recombinant plasmids have been confirmed by DNA sequencing.

### Expression and purification

The expression plasmids were amplified in *E. coli* JM110 and then transferred into *Hfx. volcanii* WFD11 [31]. The transformed cells were plated on 18% MGM agar plates containing 1% skim milk and 0.3 µg/ml novobiocin, and incubated at 37°C for 7 days. Successful *Hfx. volcanii* WFD11 transformants were identified by formation of halos around colonies [6].

*Hfx. volcanii* WFD11 transformants harboring different expression plasmids were subsequently cultivated in 18% MGM liquid media containing 0.4 µg/ml novobiocin 37°C. The culture was harvested at the late exponential phase (OD_600_ = ∼1.5). The culture supernatant containing secreted recombinant enzyme was recovered by centrifugation at 10,000× *g* for 10 min. The remaining cells were washed 3 times with buffer A (50 mM Tris-HCl, 10 mM CaCl_2_, 3 M NaCl, pH 8.0), followed by sonication in the same buffer. The soluble and insoluble cellular fractions were separated by centrifugation at 13,400× *g* for 10 min, and were used for expression analysis. The insoluble cellular fraction was subjected to 3 successive washes with buffer A before use.

The culture supernatant containing SptAH was applied to a Ni-charged Chelating Sepharose Fast Flow resin (Amersharm Biosciences, Sweden) column (1.6×20 cm) equilibrated with buffer A. After washing the column with buffer A containing 20 mM imidazole, the Ni-bound SptAH was eluted with the same buffer containing 200 mM imidazole, and the eluted fraction was dialyzed against buffer A at 4°C. The purified SptAH was supplemented with 10 mM dithiothreitol (DTT), and then incubated at 40°C for 90 min, until SptAH had fully converted to SptAΔC110 (SptA minus the CTE) and SptAC110 (the processed CTE). The sample was subsequently re-applied to a Ni-charged Chelating Sepharose Fast Flow column. The wash fractions containing SptAΔC110 were collected and His-tagged SptAC110 was eluted from the column as described above. The samples were dialyzed against buffer A at 4°C and used as the purified proteins. The protein sample was concentrated with a Microcon YM-3 centrifugal filter (Amicon) as needed. The protein concentration was determined using a Bio-Rad Bradford protein assay kit (Bio-Rad Laboratories, Inc.) with BSA as the standard.

### Preparation of SptA precursor and the isolated CTE domain (CTE*)

The gene fragments encoding SptA precursor and the CTE* (from Thr287 to Arg 413, Fig. 1) were amplified from the genomic DNA of *Natrinema* sp. J7 with primer pairs sptA-ATG1 and sptA-R2, sptAC125-F and sptAC125H-R (Supplemental Table 1), respectively, and then ligated into the *Nde*I-*Hind*III sites of pET26b to construct expression vectors pET26-*sptAH* and pET26-*sptAC125H*. Then, *E. coli* BL21(DE3) cells individually containing the expression vectors were cultured in LB medium supplemented with 1% glucose and 30 µg/ml kanamycin at 37°C until the OD_600_ reached approximately 0.6. The target proteins were induced with 0.4 mM isopropyl β-D-1-thiogalactopyranoside (IPTG), and the cultivation was continued at 37°C for 4 h. The cells were washed with buffer B (50 mM Tris-HCl, 10 mM CaCl_2_, 0.15 M NaCl, pH 8.0), and then suspended in buffer C (50 mM Tris-HCl, 10 mM CaCl_2_, 8 M Urea, pH 8.0) and disrupted by sonication. The supernatant was collected by centrifugation at 13,000× *g* for 10 min at 4°C and then subjected to a Ni-charged Chelating Sepharose Fast Flow column for purification. After washing the column with buffer C containing 40 mM imidazole, the Ni-bound protein was eluted with buffer C containing 200 mM imidazole. The purified protein was dialyzed against buffer A or B before use.

### Enzyme activity assay

Unless otherwise indicated, (azo)caseinolytic activity of the enzyme was measured at 40°C for 30 min in 400 µl of reaction mixture containing 0.25% (w/v) azocasein (Sigma A2765, MW = 23600) or 1% (w/v) casein (Sigma) and 200 µl of enzyme sample in buffer A. The reaction was terminated by the addition of 400 µl of 40% (w/v) trichloroacetic acid (TCA) into the reaction mixture. After incubating the mixture at room temperature for 15 min, it was centrifugated at 13,400× *g* for 10 min, and the absorbance of the supernatant was measured in a 1 cm cell at 366 nm (azocasein) or 280 nm (casein). One unit (U) of caseinolytic activity was defined as the amount of enzyme required to increase the absorbance units by 0.01 per minute under these reaction conditions. One unit of azocaseinolytic activity was defined as the amount of enzyme required to release 1 µg of soluble azopeptide per minute. The specific absorption coefficient (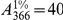) of the azocasein solution was calculated by measuring its absorption after total digestion [32].

The enzymatic activity towards azo dye-impregnated collagen (azocoll), keratin-azure or elastin-orcein (Sigma) was measured as described previously [33], except that the reaction was performed at 40°C for 60 min in buffer A.

The enzymatic activity on suc-AAPF-pNA (Sigma) was measured at 40°C in buffer A containing 0.2 mM substrate unless indicated otherwise. The activity was recorded by monitoring the initial velocity of suc-AAPF-pNA hydrolysis at 410 nm in a thermostated spectrophotometer (Cintra 10e, GBC, Australia), calculated on the basis of an extinction coefficient for *p*-nitroaniline of 8,480 M^−1^ cm^−1^ at 410 nm [34]. One unit (U) of enzyme activity was defined as the amount of enzyme that produced 1 µmol of pNA per minute under the assay conditions.

### Measurement of kinetic parameters

Suc-AAPF-pNA was used as the substrate to determine the kinetic parameters of the enzymes. Kinetic parameters were calculated from the initial velocity of hydrolysis at 40°C in buffer A, with a substrate concentration range of 0.1–2.5 mM. *K*_m_ and *k*_cat_ values were obtained by using the nonlinear regression Table Curve 2D software (Jandel Scientific, version 5.0).

### SDS-PAGE, N-terminal sequencing and immunoblot analysis

The SDS-PAGE was carried out employing glycine-Tris [35] or Tricine-Tris buffer systems [36]. The N-terminal sequencing, preparation of antisera against SptA precursor and immunoblot analysis were carried out as described previously [33].

### Far-UV circular dichroism (CD) analysis

The far-UV CD spectra were measured on a Jasco J-810 spectropolarimeter (Jasco Corporation, Tokyo, Japan) at a protein concentration of 0.2 mg/ml using a 1 mm path length cell. In the cases of SptAH and SptAΔC110, the enzymes were first inactivated by treatment with 10 mM phenylmethanesulfonyl fluoride (PMSF) in buffer A for 2 h at 30°C, followed by dialysis overnight against buffer A alone at 4°C. Since the dynode voltage exceeded 0.6 kV below 205 nm due to the high NaCl concentration, CD spectra were recorded from 205 to 260 nm. After subtracting a corresponding solvent spectrum, the sample spectrum was converted to the mean residue ellipticity [θ] (deg· cm^2^· dmol^−1^) by using mean residue molecular weights of 103.234 Da (SptAH), 101.155 Da (SptAΔC110), and 108.855 Da (CTE*).

### Binding assay on insoluble substrates

Azocoll, keratin-azure, elastin-orcein and type I collagen (Sigma) were used as the insoluble substrates. Substrate-binding assays were performed according to the batch method of Itoi et al. [21]. Briefly, using either buffer A or buffer B, 5 mg of the insoluble substrate was washed with ice-chilled buffer 3 times then suspended in 200 µl of buffer and kept at 0°C for 60 min. After centrifugation at 10,000× *g* for 5 min, the pellet was suspended in 80 µl of buffer containing enzyme at a concentration of 15 µg/ml. Unless otherwise indicated, the mixture was incubated at 0°C for 60 min, then the soluble fraction was recovered by centrifugation as described above and subjected to SDS-PAGE analysis. Protein bands were quantitated by densitometry using GeneTools software (Syngene).

## Supporting Information

Figure S1 Superimposition of SptA (*A*) and its CTE (*B*) with their homologues. The structure models of SptA and its CTE (*green*) were generated by automated homology modeling using SWISS-MODEL (http://swissmodel.expasy.org), with the kexin-like serine protease (ASP) (*cyan*) from *A. sobria* (PDB code 3HJR) and the CBD (*yellow*) of the collagenase from *C. histolyticum* (PDB code 1NQD) as the templates, respectively. The figure was prepared by PyMol (http://www.pymol.org). *A*, the active site residues Asp38, His79 and Ser232 of SptA are indicated by *D*, *H* and *S*, respectively. *B*, the lower panel shows the sequence alignment of the CTE (AAX19896), the CBD (BAA77453) and the C-CPE of *C. perfringens* enterotoxin (AAA72120). The residues indicated with stars in the CBD or the C-CPE represent those involved in collagen or receptor binding. Arrow heads indicate the three residues shown to increase collagen-binding ability of the CBD when mutated to Ala.
(TIF)

Table S1 Oligonucleotide primers used in this study.
(DOC)

## References

[1] De Castro RE, Maupin-Furlow JA, Gimenez MI, Herrera Seitz MK, Sanchez JJ (2006). Haloarchaeal proteases and proteolytic systems.. FEMS Microbiol Rev.

[2] Madern D, Ebel C, Zaccai G (2000). Halophilic adaptation of enzymes.. Extremophiles.

[3] Ryu K, Kim J, Dordick JS (1994). Catalytic properties and potential of an extracellular protease from an extreme halophile.. Enzyme Microb Technol.

[4] Kamekura M, Seno Y, Holmes ML, Dyall-Smith ML (1992). Molecular cloning andsequencing of the gene for a halophilic alkaline serine protease (halolysin) from an unidentified halophilic archaea strain (172P1) and expression of the gene in *Haloferax volcanii*.. J Bacteriol.

[5] Kamekura M, Seno Y, Dyall-Smith M (1996). Halolysin R4, a serine proteinase from the halophilic archaeon *Haloferax mediterranei*; gene cloning, expression and structural studies.. Biochim Biophys Acta.

[6] Shi W, Tang XF, Huang Y, Gan F, Tang B (2006). An extracellular halophilic protease SptA from a halophilic archaeon *Natrinema* sp. J7: gene cloning, expression and characterization.. Extremophiles.

[7] De Castro RE, Ruiz DM, Gimenez MI, Silveyra MX, Paggi RA (2008). Gene cloning and heterologous synthesis of a haloalkaliphilic extracellular protease of *Natrialba magadii* (Nep).. Extremophiles.

[8] Siezen RJ, Leunissen JA (1997). Subtilases: the superfamily of subtilisin-like serine proteases.. Protein Sci.

[9] Nonaka T, Fujihashi M, Kita A, Saeki K, Ito S (2004). The crystal structure of an oxidatively stable subtilisin-like alkaline serine protease, KP-43, with a C-terminal beta-barrel domain.. J Biol Chem.

[10] Foophow T, Tanaka S, Angkawidjaja C, Koga Y, Takano K (2010). Crystal structure of a subtilisin homologue, Tk-SP, from *Thermococcus kodakaraensis*: requirement of a C-terminal beta-jelly roll domain for hyperstability.. J Mol Biol.

[11] Ottmann C, Rose R, Huttenlocher F, Cedzich A, Hauske P (2009). Structural basis for Ca2+-independence and activation by homodimerization of tomato subtilase 3.. Proc Natl Acad Sci U S A.

[12] Kobayashi H, Utsunomiya H, Yamanaka H, Sei Y, Katunuma N (2009). Structural basis for the kexin-like serine protease from *Aeromonas sobria* as sepsis-causing factor.. J Biol Chem.

[13] Holyoak T, Wilson MA, Fenn TD, Kettner CA, Petsko GA (2003). 2.4 A resolution crystal structure of the prototypical hormone-processing protease Kex2 in complex with an Ala-Lys-Arg boronic acid inhibitor.. Biochemistry.

[14] Henrich S, Cameron A, Bourenkov GP, Kiefersauer R, Huber R (2003). The crystal structure of the proprotein processing proteinase furin explains its stringent specificity.. Nat Struct Biol.

[15] Kim DW, Matsuzawa H (2000). Requirement for the COOH-terminal pro-sequence in the translocation of aqualysin I across the cytoplasmic membrane in *Escherichia coli*.. Biochem Biophys Res Commun.

[16] Bian Y, Liang X, Fang N, Tang XF, Tang B (2006). The roles of surface loop insertions and disulfide bond in the stabilization of thermophilic WF146 protease.. FEBS Lett.

[17] Perry LJ, Wetzel R (1984). Disulfide bond engineered into T4 lysozyme: stabilization of the protein toward thermal inactivation.. Science.

[18] Greenfield N, Fasman GD (1969). Computed circular dichroism spectra for the evaluation of protein conformation.. Biochemistry.

[19] Griffin HL, Greene RV, MA C (1992). Isolation and characterization of an alkaline protease from the marine shipworm bacterium.. Current Microbiol.

[20] Capiralla Hemachander, Hiroi Tetsuya, Hirokawa Takahiko, Maeda S (2002). Purification and characterization of a hydrophobic amino acid-specific endopeptidase from *Halobacterium halobium* S9 with potential application in debittering of protein hydrolysates.. Process Biochemistry.

[21] Itoi Y, Horinaka M, Tsujimoto Y, Matsui H, Watanabe K (2006). Characteristic features in the structure and collagen-binding ability of a thermophilic collagenolytic protease from the thermophile *Geobacillus collagenovorans* MO-1.. J Bacteriol.

[22] Van Itallie CM, Betts L, Smedley JG, McClane BA, Anderson JM (2008). Structure of the claudin-binding domain of Clostridium perfringens enterotoxin.. J Biol Chem.

[23] Wilson JJ, Matsushita O, Okabe A, Sakon J (2003). A bacterial collagen-binding domain with novel calcium-binding motif controls domain orientation.. EMBO J.

[24] Zhou A, Martin S, Lipkind G, LaMendola J, Steiner DF (1998). Regulatory roles of the P domain of the subtilisin-like prohormone convertases.. J Biol Chem.

[25] Kim JS, Kluskens LD, de Vos WM, Huber R, van der Oost J (2004). Crystal structure of fervidolysin from *Fervidobacterium pennivorans*, a keratinolytic enzyme related to subtilisin.. J Mol Biol.

[26] Brown CK, Gu ZY, Matsuka YV, Purushothaman SS, Winter LA (2005). Structure of the streptococcal cell wall C5a peptidase.. Proc Natl Acad Sci U S A.

[27] Kagawa TF, O'Connell MR, Mouat P, Paoli M, O'Toole PW (2009). Model for substrate interactions in C5a peptidase from *Streptococcus pyogenes*: A 1.9 A crystal structure of the active form of ScpA.. J Mol Biol.

[28] Watanabe K (2004). Collagenolytic proteases from bacteria.. Appl Microbiol Biotechnol.

[29] Nishi N, Matsushita O, Yuube K, Miyanaka H, Okabe A (1998). Collagen-binding growth factors: production and characterization of functional fusion proteins having a collagen-binding domain.. Proc Natl Acad Sci U S A.

[30] Sarkar G, Sommer SS (1990). The “megaprimer” method of site-directed mutagenesis.. Biotechniques.

[31] Cline SW, Lam WL, Charlebois RL, Schalkwyk LC, Doolittle WF (1989). Transformation methods for halophilic archaebacteria.. Can J Microbiol.

[32] Wang CC, Houng HC, Chen CL, Wang PJ, Kuo CF (2009). Solution structure and backbone dynamics of streptopain: insight into diverse substrate specificity.. J Biol Chem.

[33] Cheng G, Zhao P, Tang XF, Tang B (2009). Identification and characterization of a novel spore-associated subtilase from Thermoactinomyces sp. CDF.. Microbiology.

[34] DelMar EG, Largman C, Brodrick JW, Geokas MC (1979). A sensitive new substrate for chymotrypsin.. Anal Biochem.

[35] King J, Laemmli UK (1971). Polypeptides of the tail fibres of bacteriophage T4.. J Mol Biol.

[36] Schagger H, von Jagow G (1987). Tricine-sodium dodecyl sulfate-polyacrylamide gel electrophoresis for the separation of proteins in the range from 1 to 100 kDa.. Anal Biochem.

