# Supplementary figures and images for: Functional Insight into the C-Terminal Extension of Halolysin SptA from Haloarchaeon Natrinema sp. J7
Source: PLoS One. 2011 Aug 19;6(8):e23562. doi: 10.1371/journal.pone.0023562 (PMC3158780; doi:10.1371/journal.pone.0023562)

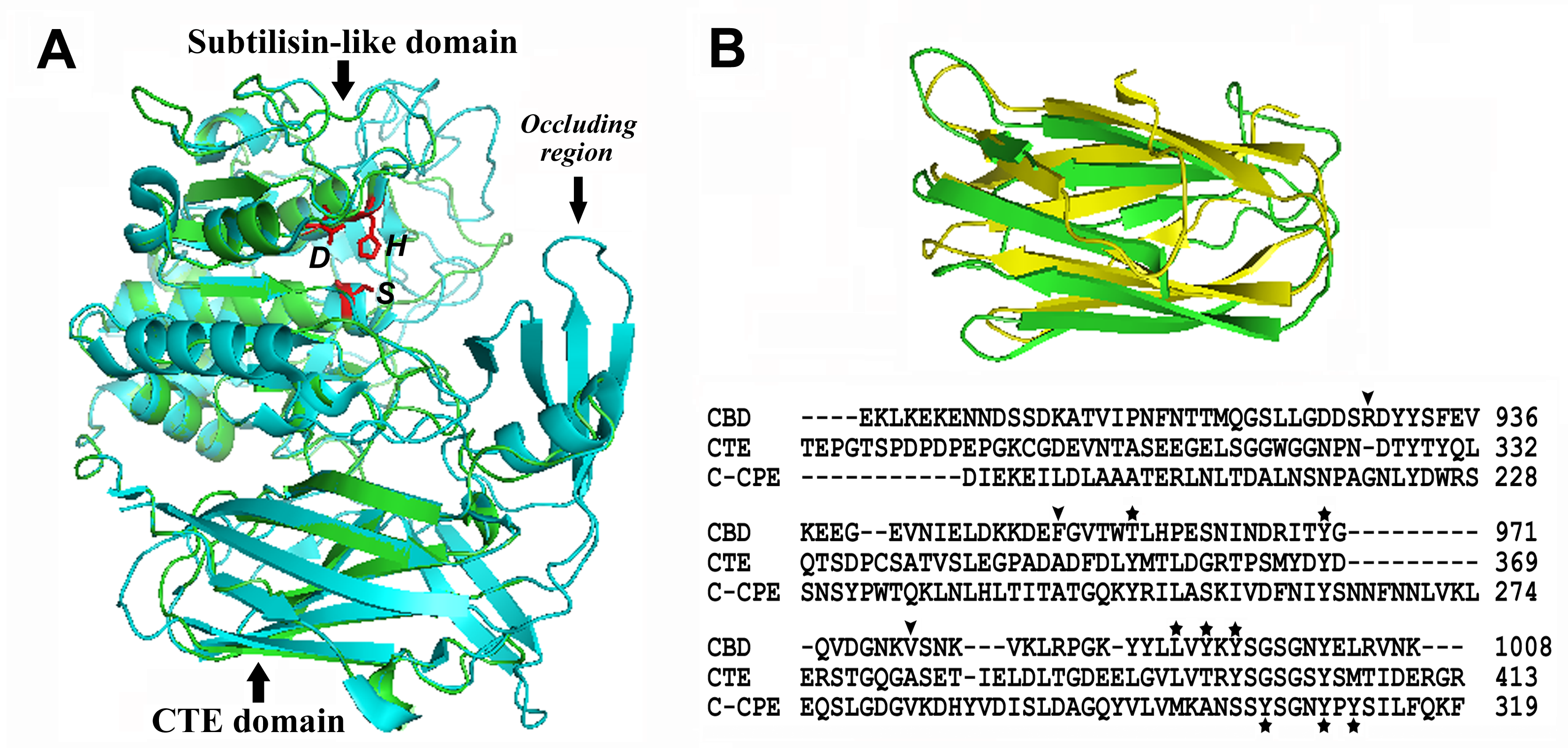

Supplement: Figure S1 — Superimposition of SptA (A) and its CTE (B) with their homologues. The structure models of SptA and its CTE (green) were generated by automated homology modeling using SWISS-MODEL (http://swissmodel.expasy.org), with the kexin-like serine protease (ASP) (cyan) from A. sobria (PDB code 3HJR) and the CBD (yellow) of the collagenase from C. histolyticum (PDB code 1NQD) as the templates, respectively. The figure was prepared by PyMol (http://www.pymol.org). A, the active site residues Asp38, His79 and Ser232 of SptA are indicated by D, H and S, respectively. B, the lower panel shows the sequence alignment of the CTE (AAX19896), the CBD (BAA77453) and the C-CPE of C. perfringens enterotoxin (AAA72120). The residues indicated with stars in the CBD or the C-CPE represent those involved in collagen or receptor binding. Arrow heads indicate the three residues shown to increase collagen-binding ability of the CBD when mutated to Ala. (TIF) [file pone.0023562.s001.tif]
