# Supplementary material for: Functional Insight into the C-Terminal Extension of Halolysin SptA from Haloarchaeon Natrinema sp. J7
Source: PLoS One. 2011 Aug 19;6(8):e23562. doi: 10.1371/journal.pone.0023562 (PMC3158780; doi:10.1371/journal.pone.0023562)
Supplement: Table S1 — Oligonucleotide primers used in this study. (DOC) [file pone.0023562.s002.doc]

**Table S1. Oligonucleotide primers used in this study.**

Italicized sections are restriction enzyme sites. Mutated nucleotides are boxed. Underlined sections indicate the 6 × His-tag encoding DNA sequences.

| Primer | Oligonucleotide sequence |
| --- | --- |
| sptA-ATG1  sptA-R1  sptA-R2  sptAH-R  sptAΔC125-R  sptAΔC76-R  sptAC103S-F  sptAC103S-R  sptAC154S-F  sptAC154S-R  sptAC303S-F  sptAC338S-F  sptAC125-F  sptAC125H-R | 5’-GCG*CATATG*TTTAGGAAGAATTTAATAGCGTGCTGGA-3’  5’-CAA*CCATGG*CTATCGACCGCGTTCGTCGATAG-3’  5’-GTG*AAGCTT*TCGACCGCGTTCGTCGATAG-3’  5’-AGC*CCATGG*CTAATGATGATGGTGGTGATGTCGACCGCGTTCGTCGATAGT-3’  5’-GGT*CCATGG*CTAGGTGGTGACGGCATTGCCG-3’  5’-GGC*CCATGG*CTAGGGGTCGGACGTCTGAAGCTG-3’  5’-GGTATTTCGAACTCGTCGCTGCTCTCG-3’  5’-CGAGAGCAGCGACGAGTTCGAAATACC-3’  5’-GATGCGGGAGGCCTCGGAGTACGCCGCC-3’  5’-GGCGGCGTACTCCGAGGCCTCCCGCATC-3’  5’-GGAGCCCGGTAAGAGCGGCGACGAGGTC-3’  5’-GACGTCCGACCCCTCGAGTGCCACCGTC-3’  5’-GGGGG*CATATG*GAACCGGGTACCTCTCCG-3’  5’-GGG*AAGCTT*CTAGTGGTGGTGGTGGTGGTGTCGACCGCGTTCGTCGATAGTC-3’ |
